# Empty nose syndrome: new insights from a CFD approach

**DOI:** 10.1007/s00405-024-09122-w

**Published:** 2024-12-11

**Authors:** Francisco Esteban-Ortega, Lina Rosique-López, Jaime A. Ochoa-Ríos, Rafael Rodríguez-Romero, Manuel A. Burgos-Olmos

**Affiliations:** 1 Department of Surgery, School of Medicine, University of Seville, Seville, Spain; 2 Department of Otolaryngology, Hospital Universitario Virgen del Rocío, Seville, Spain; 3 Department of Otolaryngology, Hospital General Universitario Morales Meseguer, Murcia, Spain; 4 Department of Otolaryngology, Hospital Viamed, Seville, Spain; 5 Department of Radiology, Hospital Viamed, Seville, Spain; 6 Departament of Ingeniería Térmica y de Fluidos, Universidad Politécnica de Cartagena, Campus Muralla del Mar, C/Doctor Fleming, s/n, 30202 Cartagena, Murcia Spain

**Keywords:** Empty Nose Syndrome (ENS), Airflow resistance, Airflow symmetry, Computational Fluid Dynamics (CFD), Nasal cavity, Patient classification, Clinical presentation, Diagnostic criteria

## Abstract

**Objectives:**

Empty Nose Syndrome (ENS) is a debilitating condition which usually arises after aggressive turbinate reduction. However, objective tests to help in the diagnosis of this condition are lacking. Accurate diagnosis of ENS patients is critical for effective diagnosis and treatment. The article's objectives are to utilize computational fluid dynamics (CFD) to analyze nasal airflow resistance and symmetry in suspected ENS patients, classify them into distinct groups based on CFD data, and demonstrate the potential of CFD analysis in refining ENS diagnosis and guiding individualized treatment strategies.

**Methods:**

This study involved 48 patients diagnosed of ENS. However, we only considered those patients with documented prior turbinate surgery (eventually plus septal surgery), CT scan with signs of prior surgery, and a history of ENS with symptoms included in the ENS Q6. We employed computational fluid dynamics (CFD) to analyze nasal airflow resistance and symmetry. Patients were classified into three groups based on their CFD data: low resistance and normal symmetry, evident asymmetry, and normal CFD parameters.

**Results:**

Half of patients (24 out of 48) were found in the low resistance and normal symmetry group, indicating ‘typical’ ENS. A smaller group (8) exhibited evident asymmetry, suggesting unilateral ENS or failure of previous surgery. Finally, 16 patients whose CFD parameters are inside the normal range of flow and resistance were classified in the normal breathing group.

**Conclusions:**

Our findings highlight the value of CFD analysis in classifying ENS patients based on airflow characteristics, as CFD analysis seems helpful in refining the diagnosis of ENS. This classification system can potentially aid in tailoring individual treatment strategies and improving patient outcomes. Further research is necessary to validate these results and explore the clinical implications of different ENS subgroups.

**Level of evidence:**

Level 4 [1].

## Introduction

Empty Nose Syndrome (ENS) is a rare and complex nasal disorder that has remained a subject of controversy in the field of otolaryngology since its first description by Eugene Kern and Monika Stenkvist in 1994 [2]. The condition typically arises after nasal surgery, most commonly inferior turbinate reduction, and is characterized by a paradoxical nasal obstruction despite a widened nasal cavity and reduced nasal resistance [3]. Although the precise pathophysiology of ENS remains elusive, recent advances in our understanding of nasal airflow dynamics and sensory perception have shed light on the possible underpinnings of this enigmatic condition [4].

The diagnosis of ENS poses significant challenges due to the absence of reliable objective tests. In fact, Houser states that ‘it is difficult to diagnose ENS because there are no reliable objective tests’ and many patients have this diagnosis based only in their symptoms and the record of a prior turbinate surgery [5]. Common symptoms of ENS include dryness, suffocation, nasal crusting, and also nasal obstruction which is considered paradoxical as the nasal cavities are typically patent. Moreover, some patients develop aprosexia nasalis, an obsession with nasal airflow and suffocation, leading to difficulties in concentrating or managing daily life activities [6]. This has prompted some researchers to suggest that treating ENS as a somatic symptom disorder may benefit these patients [7]. Suggested tools to help in diagnosis are ENSQ6 questionnaire [8], office cotton test [9] and submucosal injection of volume fillers [10].

In addition to the diagnostic challenges, management of ENS remains difficult and often requires a tailored approach for each individual patient. Treatment options range from conservative measures such as humidification and nasal cavity cleaning to more invasive surgical procedures aimed at reconstructing the nasal airway in severe cases [11]. The primary goals of endonasal repair surgery are to reduce nasal cavity volume, increase airflow resistance, and redirect airflow away from the surgical site towards non-operated or healthier regions of the nose [8]. Given these objectives, it is crucial to have an objective technique to analyze these parameters before initiating treatment.

Computational Fluid Dynamics (CFD) has emerged as a promising tool for investigating nasal airflow patterns and simulating the potential outcomes of various treatment strategies [12, 13]. CFD analysis allows for the evaluation of airflow and resistance parameters within the nasal cavity and can provide valuable insights into the functional consequences of different surgical approaches. In this context, virtual surgery simulations can be performed to predict the outcomes of various treatment options and to aid in clinical decision-making [14–16].

In our practice, we have found the Flowgy© software [17] to be an invaluable tool for planning functional nasal surgery and refining both the diagnosis and treatment options in challenging cases such as ENS patients. The ability to visualize and analyze airflow patterns and resistance within the nasal cavity can not only facilitate the identification of ENS subtypes but also inform patient-specific management strategies that maximize therapeutic outcomes.

In this study, we aim to explore the utility of CFD analysis using Flowgy© software in the diagnosis and management of ENS, with a focus on the identification of distinct subgroups of patients based on their nasal airflow patterns and resistance parameters. By gaining a deeper understanding of the complex interplay between nasal structure and function in ENS patients, we hope to contribute to the development of more effective diagnostic and therapeutic approaches for this challenging condition.

## Patients and methods

### Study population and data collection

The present study enrolled 48 patients diagnosed with Empty Nose Syndrome (ENS) at various medical facilities. In this study, our patient cohort was compiled from different countries. All participants in this study are of Caucasian ethnicity. This demographic detail is vital relevant for interpreting the findings within the context of potential ethnic or geographical variations in ENS prevalence or manifestation. Empty nose syndrome Diagnosis was considered valid when documentation of prior nasal surgery (mainly turbinoplasty with or without septoplasty) was provided, signs of prior surgery in the computed tomography (CT) scan were evident, and patients recorded ENS Q6 scores with at least 11 points (see Table 2).The primary method of patient enrollment was through global email correspondence. The demographic and clinical data of the patients, such as age, gender, medical history, and details of prior nasal surgeries, were collected and analyzed. The study involved the retrospective selection and anonymization of CT scans with only age and sex information collected for the participants. Although the study was retrospective in nature, the protocol was reviewed and approved by the Institutional Review Board (or Ethics Committee) of the Authors' Institution to ensure appropriate data handling and maintenance of patient confidentiality (reference number CEI23_005).

### CT scan analysis

CT scans from the patients were analyzed using Flowgy© software [17], which provided a comprehensive assessment of nasal airflow and resistance parameters. This software is a tool that integrates features from previous software versions, such as MeComLand©, DigBody©, and NoseLand© [18], and facilitates the execution of the Computational Fluid Dynamics (CFD) workflow. The established protocol for the CFD workflow, as described by Burgos et al. [19], was followed in this study.

### CFD workflow

The following delineates the systematic approach employed for analyzing nasal airflow using Computational Fluid Dynamics:

1. **Segmentation and Three-Dimensional Model Construction from CT scans**: CT image data underwent a segmentation process, creating a comprehensive three-dimensional (3D) representation, encompassing facial structures, nasal airways, paranasal sinuses, nasopharynx, and a strategically devised atmospheric boundary region (Fig. 1). This boundary zone was instrumental in determining the most physiologically accurate point of air entrance during inhalation, emulating ambient atmospheric conditions. Segmentations techniques were grounded on Binary Thresholding filters, applying distinct thresholds, quantified in Hounsfield Units (HU), with HU values spanning from 350 to 3024 [20].

**Fig. 1:**
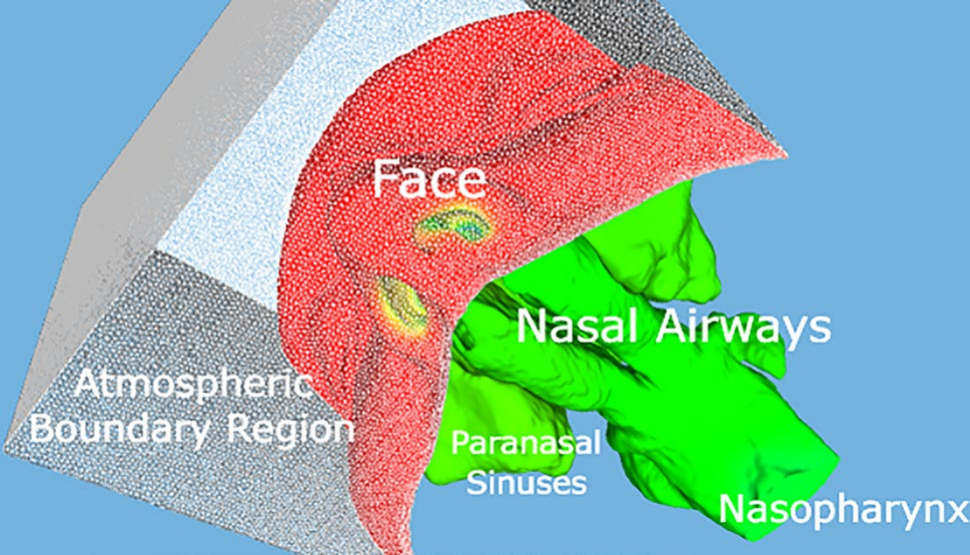
Three-dimensional representation of the nasal cavity architecture derived from one subject amongst the total of 48
2. **Discretization and Computational Mesh Generation**: 3D constructs underwent a discretization process, resulting in the formation of computational meshes. The mesh count in this study fluctuated between 7 and 15 million tetrahedral cells, a range chosen to assure that results were not sensitive to mesh size.
3. **Airflow Simulation via the Navier‐Stokes Equations**: Navier–Stokes equations were discretized over the computational mesh to facilitate airflow simulation. All simulations exclusively targeted the inhalation phase, with a focus on compressible, stable, and laminar flow conditions. To induce a volumetric airflow rate (~ 15 L/min) that maintained laminar flow, a patient-specific pressure gradient was instituted between the atmosphere and the nasopharynx [21]. The boundary conditions dictated a no-slip scenario on the wall surfaces. Ambient and internal nasal temperatures were designated at 21°C and 36.5°C, respectively, while relative humidity values at these sites were set at 20% and 100%, respectively. A meticulous grid convergence analysis, aligned with methodologies prescribed by Burgos et al. [22], was executed to validate the numerical simulations.
4. **Post-simulation Data Analysis**: Flow-related metrics requisite for the calculation of two crucial dimensionless parameters (R and ϕ) were extracted from selected cuts on the computational mesh. Figure 2 depicts the key sections of the computational domain utilized for post-processing the CFD simulation results to derive the dimensionless resistance (R) and symmetry (φ) parameters. Specifically, the computational domain was bounded by a rectangular region representing the surrounding atmosphere and a planar cross-section at the nasopharynx to model the airflow outlet. Additionally, specific planar cross-sections were defined at the right nostril, left nostril and the choana. During post-processing, relevant flow variables including local velocities, pressures, and mass flow rates were extracted and sampled from these segments of the mesh. The cross-sectional plane locations enabled quantification of the flow rate distribution between the left and right sides and the choana, which is critical for determining the symmetry parameter φ. Additionally, the pressure drop between the atmosphere and choana was calculated, which is directly related to the overall nasal resistance parameter R.

**Fig. 2:**
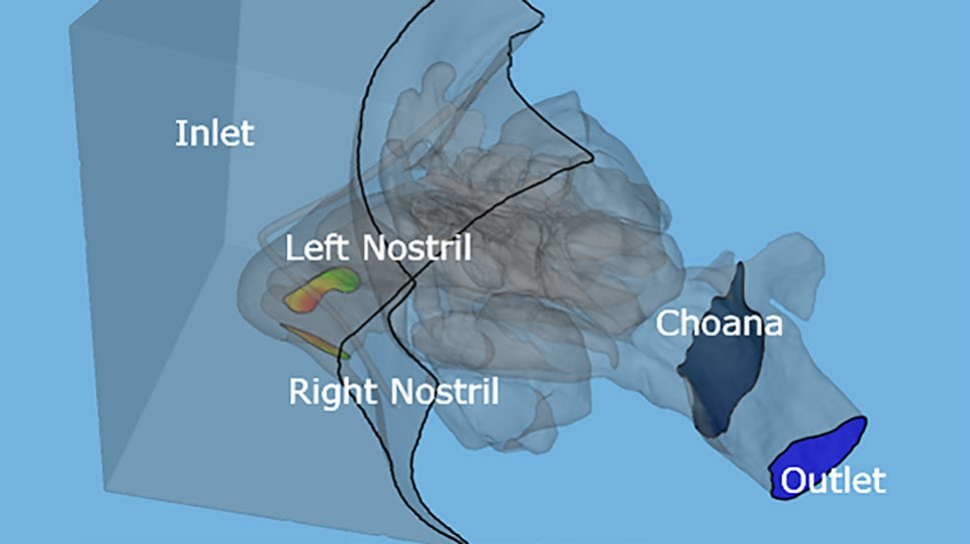
The graphic representation identifies specific zones employed to compute the dimensionless parameters ϕ and R: including right nostril, left nostril, choana, and the entry and exit zones of the computational domain

### Dimensionless parameters in nasal airflow analysis

Sanmiguel‐Rojas et al., in 2018 [23], introduced two dimensionless parameters, the symmetry (ϕ) and resistance (R), which have been pivotal in analyzing and categorizing the 48 patients diagnosed with Empty Nose Syndrome (ENS). These metrics, birthed from CFD simulations, shed light on the intrinsic aerodynamics within the nasal passages, augmenting our grasp of airflow mechanics.

- **Symmetry Parameter (ϕ)**: The symmetry index, ϕ, works as an indicator of bilateral airflow balance within the nasal domain. A ϕ value nearing unity implies equitable airflow distribution across both nostrils. A deviation from this norm can point to potential nasal blockages or associated pathologies. The relevance of ϕ lies in its ability to convey the functional value of the nasal architecture, with ENS patients potentially exhibiting varying asymmetry levels, traceable to symptom severity or past surgical interventions.
- **Resistance Parameter (R)**: Symbolized as R, this metric encapsulates the collective nasal resistance to airflow. It's imperative to underscore that nasal resistance is essential in determining nasal passage functionality. An elevated resistance could throttle airflow, manifesting as respiratory challenges and symptoms related to different nasal conditions. The R metric holistically assesses the nasal passage's resistance profile, encompassing all contributory elements like structural modifications, inflammation, or other resistance-augmenting factors.

### K-means clustering analysis: in-depth technical details

K-means clustering analysis [24, 25] is an unsupervised machine learning algorithm that aims to partition a dataset into distinct groups or clusters based on the similarity of the data points. In this study, K-means clustering was applied to a dataset consisting of 48 ENS patients, with the objective of identifying distinct patient groups based on their R and ϕ values, which are dimensionless parameters representing nasal airflow characteristics.

Algorithm and Initialization: The K-means algorithm starts selecting K initial cluster centroids, where K is a predetermined number of clusters. In this study, K was set to 3, resulting in three patient groups (Group 1, Group 2, and Group 3). The initial centroids can be selected randomly or through various initialization strategies, such as the K-means + + method [26], which aims to improve the convergence speed and clustering results.

## Results

### Patient characteristics

A total of 48 patients with a diagnosis of ENS were included in this study, with a predominance of male patients (n = 40, 83.3%) and a smaller proportion of female patients (n = 8, 16.7%). The age of the patients ranged from 18 to 65 years, with a mean age of 41.2 ± 12.7 years. Most patients had a history of turbinate reduction surgery (n = 42, 87.5%), while the remaining patients had undergone septoplasty plus turbinoplasty (n = 6, 12.5%). Length of ENS symptoms varied widely among the patients, ranging from 6 months to 10 years, with a median duration of 3 years.

### CFD results and classification of ENS patients

**Fig. 3:**
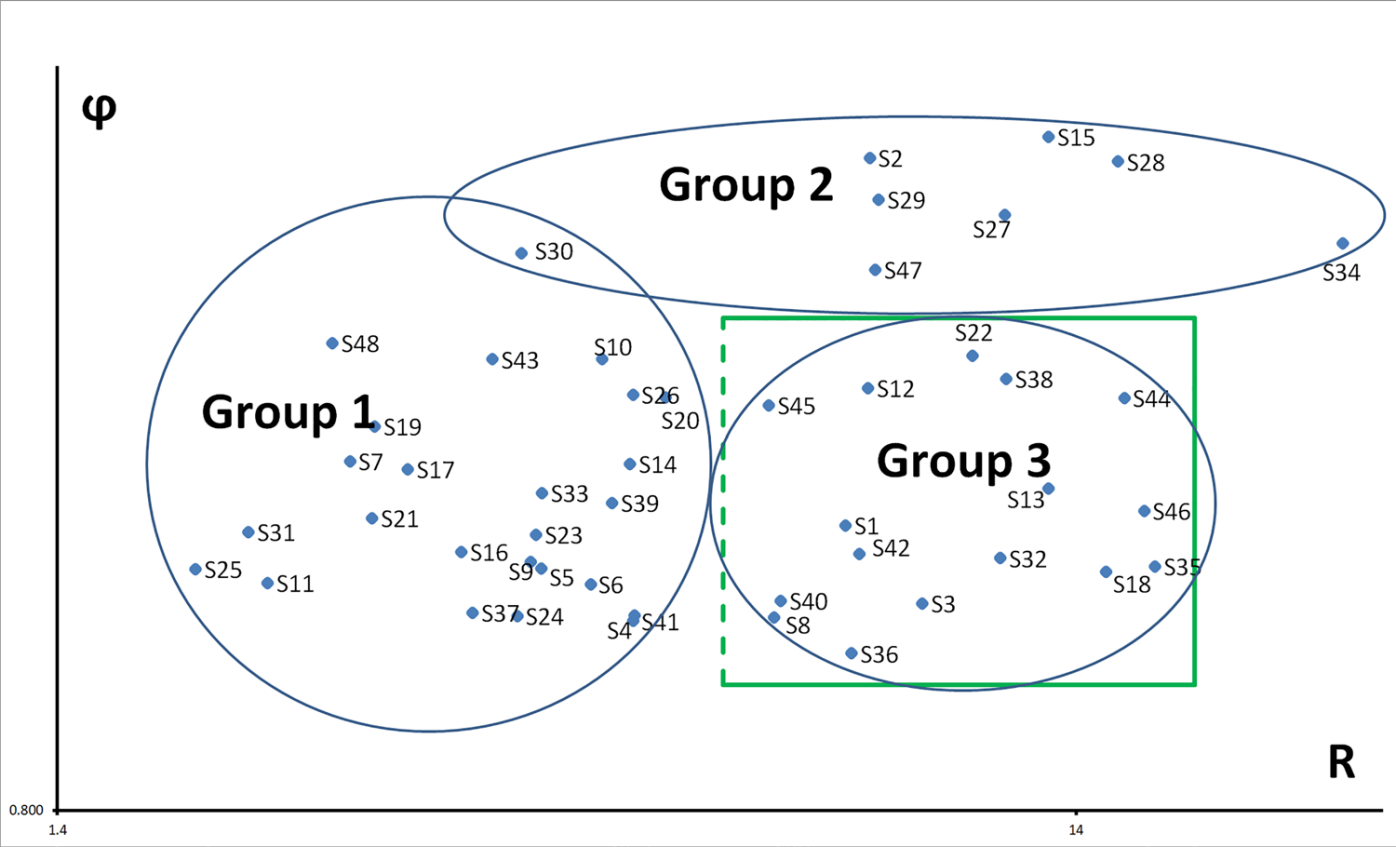
Graphical representation on Cartesian axes of the non-dimensional ϕ and R parameters for the 48 patients, grouped by the cluster they belong to

Based on the CFD analysis, patient’s CFD data can be displayed as follows in the Fig. 3. According to the calculated R and ϕ parameters and the K-means clustering analysis, the patients were classified into three distinct groups (Table 1):

**Table 1:** Group classification by k-means algorithm

Group 1	24 subjects	‘typical’ ENS
Group 2	8 subjects	‘atypical’ ENS
Group 3	16 subjects	‘Normal Airflow ENS’/normal parameters
Total	48	

The ‘typical’ ENS group consisted of patients whose CFD parameters with resistance values below normal levels, indicating a high likelihood of ENS. The 'atypical' ENS group included patients with a significant asymmetry in the flow between the two nasal cavities. Finally, the 'Normal Airflow ENS' group comprised patients whose CFD parameters were within the normal range for healthy individuals [23, 27, 28].

### Stability of the K-means clustering method in the 48-patient dataset

The stability of the K-means clustering algorithm is a crucial aspect to consider when evaluating the reliability of the clustering results obtained from the 48 ENS patients. Stability refers to the consistency of the clustering results when the algorithm is applied multiple times with different initializations. A high degree of stability suggests that the identified clusters are robust and less sensitive to variations in the initial conditions.

In Fig. 3, we have illustrated the ϕ values on the ordinate axis and the R values on the abscissa axis in a scatter plot, wherein we have indicated the corresponding parameters for the patients who have been classified into each of the three groups. In this figure, we have also depicted the normality square (green) as defined by Sanmiguel-Rojas et al. (2017) [23]. Patients included in this green square have a high probability of normal nasal breathing as previously published. In Table 2, we can see the numerical values of the ϕ and R parameters for each of the 48 patients used in this study, as well as the groups they have been classified into by the K-means Clustering algorithm.

**Table 2:** Numerical values of the ϕ and R parameters for each of the 48 patients

#Patient	# Group	ϕ	R	ENSQ6	# Patient	# Group	ϕ	R	ENSQ6
S48	1	1.636	2.605	11	S34	2	25.583	25.583	14
S10	1	1.596	4.791	20	S30	2	1.878	3.993	21
S43	1	1.596	3.74	12	S15	2	2.245	13.15	15
S26	1	1.512	5.14	27	S2	2	2.172	8.778	12
S20	1	1.505	5.531	17	S28	2	2.16	15.38	24
S19	1	1.44	2.866	12	S29	2	2.037	8.961	29
S7	1	1.366	2.712	18	S27	2	1.991	11.917	20
S14	1	1.361	5.107	11	S47	2	1.831	8.889	15
S17	1	1.349	3.09	15	S13	3	1.311	13.144	11
S33	1	1.301	4.187	22	S46	3	1.266	16.324	12
S39	1	1.281	4.903	11	S1	3	1.238	8.316	15
S21	1	1.252	2.85	14	S42	3	1.185	8.574	17
S31	1	1.226	2.156	19	S32	3	1.177	11.799	18
S23	1	1.22	4.128	11	S35	3	1.163	16.735	11
S16	1	1.188	3.487	12	S18	3	1.154	14.971	16
S9	1	1.17	4.08	11	S40	3	1.103	7.177	23
S5	1	1.159	4.177	22	S3	3	1.098	9.885	14
S25	1	1.158	1.913	11	S8	3	1.075	7.076	14
S11	1	1.134	2.252	26	S36	3	1.018	8.428	19
S6	1	1.131	4.677	16	S22	3	1.605	1.605	11
S37	1	1.083	3.577	14	S38	3	1.549	1.549	11
S4	1	1.079	5.156	11	S12	3	1.527	1.527	15
S24	1	1.077	3.958	16	S44	3	1.504	1.504	12
S41	1	1.069	5.14	30	S45	3	1.487	1.487	21

## Discussion

Nasal surgery for inferior turbinates has evolved significantly over time, transitioning from more aggressive partial or total resections to the commonly performed radiofrequency volume reduction. As the number of surgical patients increases, so too do the associated complications. Empty nose syndrome (ENS) is typically described as a rare syndrome of paradoxical nasal obstruction in patients who have undergone common nasal procedures, primarily turbinate surgery [3]. These patients report persistent symptoms, often with evidence of turbinate tissue loss, but sometimes with unremarkable examinations aside from evidence of prior surgery, leading to the term "paradoxical." The poor correlation between subjective and objective findings is a defining characteristic of ENS. Surgeons who are unaware of this diagnosis may recommend additional surgery, potentially worsening the patient's condition.

While some reports describe case series in which ENS did not develop among patients who underwent turbinate excision [29], and surgeons who perform extensive surgery for nasosinusal tumors do not report this complication, many otolaryngologists are increasingly aware of the growing body of data supporting the hypothesis that changes in airflow dynamics and neurophysiology after over-excision of turbinate tissue can lead to ENS in some patients. On the other hand, a strong association between ENS patients and impaired mental health has been reported (see for review Kanjanawasee et al. 2022) [30]. Comorbidities such as hyperventilation syndrome, anxiety, and depression have been identified in a majority of ENS patients. Many ENS patients meet the criteria for somatic symptom disorder and may benefit from psychiatric treatment, including cognitive behavioral therapy [7].

Similarly to Houser, who evaluated numerous symptoms and sinus computed tomographic scans to screen for ENS using the internet as his primary source to recruit patients [5], we also recruited most of our patients online, as ENS patients are highly active on social media. While some patients are diagnosed ENS, others reside in countries that do not recognize the condition, prompting these individuals to seek help elsewhere. In all cases, diagnosis relies on clinical symptoms, as findings are generally nonspecific. The cotton test is commonly used but, in our opinion, considering the CFD studies, it offers limited assistance when deciding on surgery.

In this study, we employed CFD analysis using Flowgy© [17] to evaluate CT scans of 48 patients with a diagnosis of ENS. Our results revealed that CFD-derived parameters (R and ϕ) could help refine the diagnosis of ENS by identifying subgroups of patients with typical ENS, atypical ENS, and Normal Airflow ENS presentations. Our findings are consistent with previous literature that has demonstrated the usefulness of CFD analysis in the assessment of various nasal pathologies. Specifically, several studies have reported that CFD-derived parameters can effectively differentiate between healthy and diseased nasal airways, contributing to a more accurate diagnosis and targeted treatment strategies. In the context of ENS, our study extends this body of evidence by demonstrating the potential role of CFD analysis in identifying subgroups of patients with different ENS presentations.

This finding underscores the complexity and heterogeneity of ENS, as well as the importance of a comprehensive diagnostic approach that includes both subjective and objective assessments. For these patients, alternative diagnoses or treatment strategies may be considered, such as addressing potential psychological components of their symptoms or revising previous surgical outcomes.

An intriguing subgroup of patients consists of those with an overall normal flow but significant asymmetry when comparing both cavities, suggesting the existence of a unilateral ENS. Due to the absence of reliable objective tests to quantify these symptoms, ENS diagnosis primarily depends on subjectively reported symptomology. The Empty Nose Syndrome 6-item Questionnaire (ENS6Q) has recently been validated for identifying patients suspected of developing ENS [8]. A score of 11 out of a possible total of 30 was determined as the cut-off criterion to predict ENS. Additionally, the cotton test has been employed to aid in the diagnosis of ENS [3]. The lack of objective tests for diagnosing this rare condition seems unreasonable, and in our experience, computational fluid dynamics (CFD) analysis using Flowgy© allows for distinguishing between patients with alterations in nasal flow and those with a normal airway, as assessed by both endoscopy and CFD studies. Patients with low resistance may benefit from techniques that narrow the nasal cavity and nasal valve area, while those with Flowgy© results within the normal range may require further investigation.

One of the limitations of our study is the reliance on self-reported ENS diagnoses and symptom duration, which could be subject to recall bias or misclassification. However, we only considered those patients with documented prior turbinate surgery (eventually plus septal surgery), CT scan with signs of prior surgery, and a history of ENS with symptoms included in the ENS Q6. Additionally, our sample size was relatively small, and the study population was predominantly male. Second, from our point of view, this methodology really improves diagnosis and treatment, as it helps to localize the anatomical sites to be treated, and a prospective study currently in progress. The group 3 of patients needs further analysis as neither CT scanning or CFD analysis shows abnormalities, and probably receptors in the nasal mucosa must be investigated and/or psychiatric support will surely needed.

Future studies with larger and more diverse samples arenecessary to confirm our findings and further explore the potential applications of CFD analysis in ENS diagnosis and treatment.

Finally, considering the strong link between Empty Nose Syndrome (ENS) and mental health disorders, further investigation into ENS's psychological aspects is essential. Comprehensive analysis of the connections between ENS, anxiety, depression, and somatic symptom disorder may lead to more effective treatments addressing both the physical and psychological components of the syndrome.

Our experience shows that using computational fluid dynamics (CFD) analysis, particularly Flowgy© software, can significantly improve our understanding of ENS and contribute to better diagnosis and treatment options. By identifying distinct patient subgroups based on nasal airflow patterns and resistance parameters, we can create more targeted and personalized therapeutic approaches. Furthermore, CFD analysis allows clinicians to simulate treatment outcomes, enabling better-informed decision-making and optimizing patient care. As our knowledge of ENS evolves, it's crucial to emphasize the use of advanced technologies like CFD to enhance our comprehension of this enigmatic disorder and ultimately improve affected individuals' lives.

In conclusion, improving the understanding of ENS and its associated factors is crucial for the development of more effective diagnostic and treatment methods. By expanding the discussion of ENS to include statistical analysis, airflow dynamics, surgical techniques, and psychological factors, researchers can gain a more comprehensive understanding of the syndrome and work towards better outcomes for patients who suffer from this debilitating condition.

## References

[1] OCEBM Levels of Evidence Working Group*. “The Oxford 2011 Levels of Evidence”. Oxford Centre for Evidence-Based Medicine. http://www.cebm.net/index.aspx?o=5653* OCEBM Table of Evidence Working Group = Jeremy Howick, Iain Chalmers (James Lind Library), Paul Glasziou, Trish Greenhalgh, Carl Heneghan, Alessandro Liberati, Ivan Moschetti, Bob Phillips, Hazel Thornton, Olive Goddard and Mary Hodgkinson. Accessed Dec 10, 2024

[2] Scheithauer MO (2011) Surgery of the turbinates and “empty nose” syndrome. GMS Curr Top Otorhinolaryngol Head Neck Surg 9:Doc03. 10.3205/cto000067 10.3205/cto000067 PMC3199827 22073107

[3] Chhabra N, Houser SM (2009) The diagnosis and management of empty nose syndrome. Otolaryngol Clin N Am 42(2):311–330. 10.1016/j.otc.2009.02.001 10.1016/j.otc.2009.02.001 19328895

[4] Payne SC (2009) Empty nose syndrome: what are we really talking about? Otolaryngol Clin N Am 42(2):331–337. 10.1016/j.otc.2009.02.002. (**ix-x**) 10.1016/j.otc.2009.02.002 19328896

[5] Houser SM (2007) Surgical treatment for empty nose syndrome. Arch Otolaryngol Head Neck Surg 133(9):858–863. 10.1001/archotol.133.9.858 17875850 10.1001/archotol.133.9.858

[6] Talmadge J, Nayak JV, Yao W, Citardi MJ (2019) Management of postsurgical empty nose syndrome. Facial Plast Surg Clin N Am 27(4):465–475. 10.1016/j.fsc.2019.07.005 10.1016/j.fsc.2019.07.005 31587766

[7] Lemogne C, Consoli SM, Limosin F, Bonfils P (2015) Treating empty nose syndrome as a somatic symptom disorder. Gen Hosp Psychiatry 37(3):273.e9-273.e10. 10.1016/j.genhosppsych.2015.02.005 25754986 10.1016/j.genhosppsych.2015.02.005

[8] Velasquez N, Thamboo A, Habib A-RR, Huang Z, Nayak JV (2017) The Empty Nose Syndrome 6-Item Questionnaire (ENS6Q): a validated 6-item questionnaire as a diagnostic aid for empty nose syndrome patients. Int Forum Allergy Rhinol 7(1):64–71. 10.1002/alr.21842 27557473 10.1002/alr.21842

[9] Malik J et al (2020) The cotton test redistributes nasal airflow in patients with empty nose syndrome. Int Forum Allergy Rhinol. 10(4):539–545. 10.1002/alr.22489 31951101 10.1002/alr.22489 PMC7182493

[10] Borchard NA, Dholakia SS, Yan CH, Zarabanda D, Thamboo A, Nayak JV (2019) Use of intranasal submucosal fillers as a transient implant to alter upper airway aerodynamics: implications for the assessment of empty nose syndrome. Int Forum Allergy Rhinol 9(6):681–687. 10.1002/alr.22299 30715801 10.1002/alr.22299

[11] Coste A, Dessi P, Serrano E (2012) Empty nose syndrome. Eur Ann Otorhinolaryngol Head Neck Dis 129(2):93–97. 10.1016/j.anorl.2012.02.001 22513047 10.1016/j.anorl.2012.02.001

[12] Li C et al (2017) Computational fluid dynamics and trigeminal sensory examinations of empty nose syndrome patients: Computational and Trigeminal Studies of ENS. Laryngoscope 127(6):E176–E184. 10.1002/lary.26530 28278356 10.1002/lary.26530 PMC5445013

[13] Malik J, Otto BA, Zhao K (2022) Computational FLUID DYNAmics (CFD) modeling as an objective analytical tool for nasal/upper airway breathing. Curr Otorhinolaryngol Rep 10(1):116–120. 10.1007/s40136-021-00387-x

[14] Burgos MA, Sanmiguel-Rojas E, Del Pino C, Sevilla-García MA, Esteban-Ortega F (2017) New CFD tools to evaluate nasal airflow. Eur Arch Oto-Rhino-Laryngol 274(8):3121–3128. 10.1007/s00405-017-4611-y 10.1007/s00405-017-4611-y 28547013

[15] Burgos Olmos MA et al (2018) Cirugía virtual para pacientes con obstrucción nasal: empleo de un software basado en dinámica de fluidos (MeComLand®, Digbody® & Noseland®) para documentar parámetros objetivos de flujo y optimizar resultados quirúrgicos, Acta Otorrinolaringológica Esp. Organo Of. Soc. Esp. Otorrinolaringol. Patol. Cérv.-fac., vol. 69, n.^o^ 3 (Mayo-Junio 2018), pp. 125–133, 2018, Accedido: 21 de enero de 2024. [En línea]. Disponible en: https://dialnet.unirioja.es/servlet/articulo?codigo=6416446 10.1016/j.otorri.2017.05.005 28923473

[16] Burgos MA, Sanmiguel-Rojas E, Singh N, Esteban-Ortega F (2018) DigBody: a new 3D modeling tool for nasal virtual surgery. Comput Biol Med 98:118–125. 10.1016/j.compbiomed.2018.05.016 29787939 10.1016/j.compbiomed.2018.05.016

[17] Flowgy The most affordable, fastest and efficient nasal surgery. Accedido: 27 de diciembre de 2023. [En línea]. Disponible en: https://www.flowgy.com/. Accessed Dec 10, 2024

[18] Burgos MA et al (2018) Virtual surgery for patients with nasal obstruction: Use of computational fluid dynamics (MeComLand®, Digbody® & Noseland®) to document objective flow parameters and optimise surgical results. Acta Otorrinolaringol Esp 69(3):125–133. 10.1016/j.otorri.2017.05.005 10.1016/j.otorri.2017.05.005 28923473

[19] Burgos MA, Sanmiguel-Rojas E, del Pino C, Sevilla-García MA, Esteban-Ortega F (2017) New CFD tools to evaluate nasal airflow. Eur Arch Otorhinolaryngol 274(8):3121–3128. 10.1007/s00405-017-4611-y 28547013 10.1007/s00405-017-4611-y

[20] Bastir M, Sanz-Prieto D, Burgos-Olmos M (2021) Three-dimensional form and function of the nasal cavity and nasopharynx in humans and chimpanzees. Anat Rec 305(8):12. 10.1002/ar.24790 10.1002/ar.24790 34636487

[21] Taylor DJ, Doorly DJ, Schroter RC (2010) Inflow boundary profile prescription for numerical simulation of nasal airflow. J R Soc Interface 7(44):515–527. 10.1098/rsif.2009.0306 19740920 10.1098/rsif.2009.0306 PMC2842801

[22] Burgos MA, Sanmiguel-Rojas E, Martín-Alcántara A, Hidalgo-Martínez M (2013) Effects of the ambient temperature on the airflow across a Caucasian nasal cavity. Int J Numer Methods Biomed Eng 30(3):430–445. 10.1002/cnm.2616 10.1002/cnm.2616 24574201

[23] Sanmiguel-Rojas E, Burgos M, Del Pino C, Sevilla-García M, Esteban-Ortega F (2017) Robust non-dimensional estimators to assess the nasal airflow in health and disease. Int J Numer Methods Biomed Eng, p 101002cnm2906 2017 10.1002/cnm.2906 28574647

[24] MacQueen J (1967) Some methods for classification and analysis of multivariate observations, 1967. [En línea]. Disponible en: https://www.semanticscholar.org/paper/Some-methods-for-classification-and-analysis-of-MacQueen/ac8ab51a86f1a9ae74dd0e4576d1a019f5e654ed. Accessed Dec 10, 2024

[25] Hartigan JA, Wong MA (1979) A K-Means clustering algorithm. J R Stat Soc Ser C Appl Stat 28(1):100–108. 10.2307/2346830

[26] Arthur D, Vassilvitskii S (2007) k-means++: the advantages of careful seeding. In: Proceedings of the Eighteenth Annual ACM-SIAM symposium on Discrete algorithms, en SODA ’07. USA: Society for Industrial and Applied Mathematics, ene. 2007, pp 1027–1035

[27] Sanmiguel-Rojas E, Burgos MA, Esteban-Ortega F (2018) Nasal surgery handled by CFD tools. Int J Numer Methods Biomed Eng. 10.1002/cnm.3126 10.1002/cnm.3126 29968373

[28] Burgos MA et al (2021) Linking chronic otitis media and nasal obstruction: A CFD approach. Laryngoscope 132(6):7. 10.1002/lary.29882 10.1002/lary.29882 34585755

[29] Talmon Y, Samet A, Gilbey P (2000) Total inferior turbinectomy: operative results and technique. Ann Otol Rhinol Laryngol 109(12 Pt 1):1117–1119. 10.1177/000348940010901206 11130822 10.1177/000348940010901206

[30] Kanjanawasee D et al (2022) Empty nose syndrome pathophysiology: a systematic review. Otolaryngol-Head Neck Surg 167(3):434–451. 10.1177/01945998211052919 34665687 10.1177/01945998211052919

